# Exploring Strong Interactions in Proteins with Quantum Chemistry and Examples of Their Applications in Drug Design

**DOI:** 10.1371/journal.pone.0137113

**Published:** 2015-09-04

**Authors:** Neng-Zhong Xie, Qi-Shi Du, Jian-Xiu Li, Ri-Bo Huang

**Affiliations:** 1 State Key Laboratory of Non-food Biomass and Enzyme Technology, National Engineering Research Center for Non-food Biorefinery, Guangxi Academy of Sciences, 98 Daling Road, Nanning, Guangxi, 530007, China; 2 Life Science and Biotechnology College, Guangxi University, Nanning, Guangxi, 530004, China; 3 Gordon Life Science Institute, 53 South Cottage Road, Belmont, MA, 02478, United States of America; Indiana University School of Medicine, UNITED STATES

## Abstract

**Objectives:**

Three strong interactions between amino acid side chains (salt bridge, cation-π, and amide bridge) are studied that are stronger than (or comparable to) the common hydrogen bond interactions, and play important roles in protein-protein interactions.

**Methods:**

Quantum chemical methods MP2 and CCSD(T) are used in calculations of interaction energies and structural optimizations.

**Results:**

The energies of three types of amino acid side chain interactions in gaseous phase and in aqueous solutions are calculated using high level quantum chemical methods and basis sets. Typical examples of amino acid salt bridge, cation-π, and amide bridge interactions are analyzed, including the inhibitor design targeting neuraminidase (NA) enzyme of influenza A virus, and the ligand binding interactions in the HCV p7 ion channel. The inhibition mechanism of the M2 proton channel in the influenza A virus is analyzed based on strong amino acid interactions.

**Conclusion:**

(1) The salt bridge interactions between acidic amino acids (Glu^-^ and Asp^-^) and alkaline amino acids (Arg^+^, Lys^+^ and His^+^) are the strongest residue-residue interactions. However, this type of interaction may be weakened by solvation effects and broken by lower pH conditions. (2) The cation- interactions between protonated amino acids (Arg^+^, Lys^+^ and His^+^) and aromatic amino acids (Phe, Tyr, Trp and His) are 2.5 to 5-fold stronger than common hydrogen bond interactions and are less affected by the solvation environment. (3) The amide bridge interactions between the two amide-containing amino acids (Asn and Gln) are three times stronger than hydrogen bond interactions, which are less influenced by the pH of the solution. (4) Ten of the twenty natural amino acids are involved in salt bridge, or cation-, or amide bridge interactions that often play important roles in protein-protein, protein-peptide, protein-ligand, and protein-DNA interactions.

## Introduction

The twenty natural amino acids (abbreviated as aa), which are characterized by their unique side chains, are the building blocks of proteins and peptides [1–5]. Consequently, the interactions between aa side chains are the dominant factors in determining protein structures and interactions. These aa interactions are responsible for protein recognition [6,7], protein folding [8], protein-protein and protein-peptide interactions [9,10], protein-ligand docking [11,12], protein-DNA (or RNA) interactions [13], and information transmission by signal peptides in protein metabolism [14,15].

Due to the structural diversity of the 20 amino acid side chains, the aa side chain interactions exhibit very different energetic contributions and physical properties, which cannot be explained simply by the familiar interaction types, such as hydrogen bonds [16], van der Waals interactions [17], electrostatic interactions [18], and hydrophobic interactions [19]. In protein chemistry, hydrogen bonds that have energies in the range of 8 to 30 kJ/mol [20,21] are considered to be strong interactions. However, some aa side chain interactions in different aa pairs may be remarkably stronger than (or comparable to) hydrogen bonds.

The strong aa interactions, other than common hydrogen bonds, include salt bridge, cation-π, and amide bridge interactions, which often play important roles in protein-protein and protein-ligand interactions. For example, salt bridge interactions [22–24] play important role in the amyloid-beta plaque growth of Alzheimer’s and related diseases, and in oseltamivir–neuraminidase binding interaction of M2 proton channel in the influenza A virus [25–27]. The cation-π interactions [28,29] make main energetic contribution in the binding interaction between the ammonium group (NH_3_^+^) of amantadine and the aromatic residue Trp-21 in the p7 ion channel [30] of HCV (hepatitis C virus).

In this study the three strong aa side chain interaction types (salt bridge, cation-π, and amide bridge interactions) are theoretically studied. The energies of the three types of aa interactions are calculated in the gaseous phase and in aqueous solutions using high level quantum chemical methods and basis sets. Three typical examples of aa side chain interactions in drug design are analyzed based on the theoretical study results, including the inhibitor design targeting the neuraminidase (NA) [25] of the influenza A virus, the M2 proton channel protein [26,27] of the influenza A virus, and the p7 ion channel protein [30] of the hepatitis C virus (HCV).

## Theory and Methods

**Fig 1:**
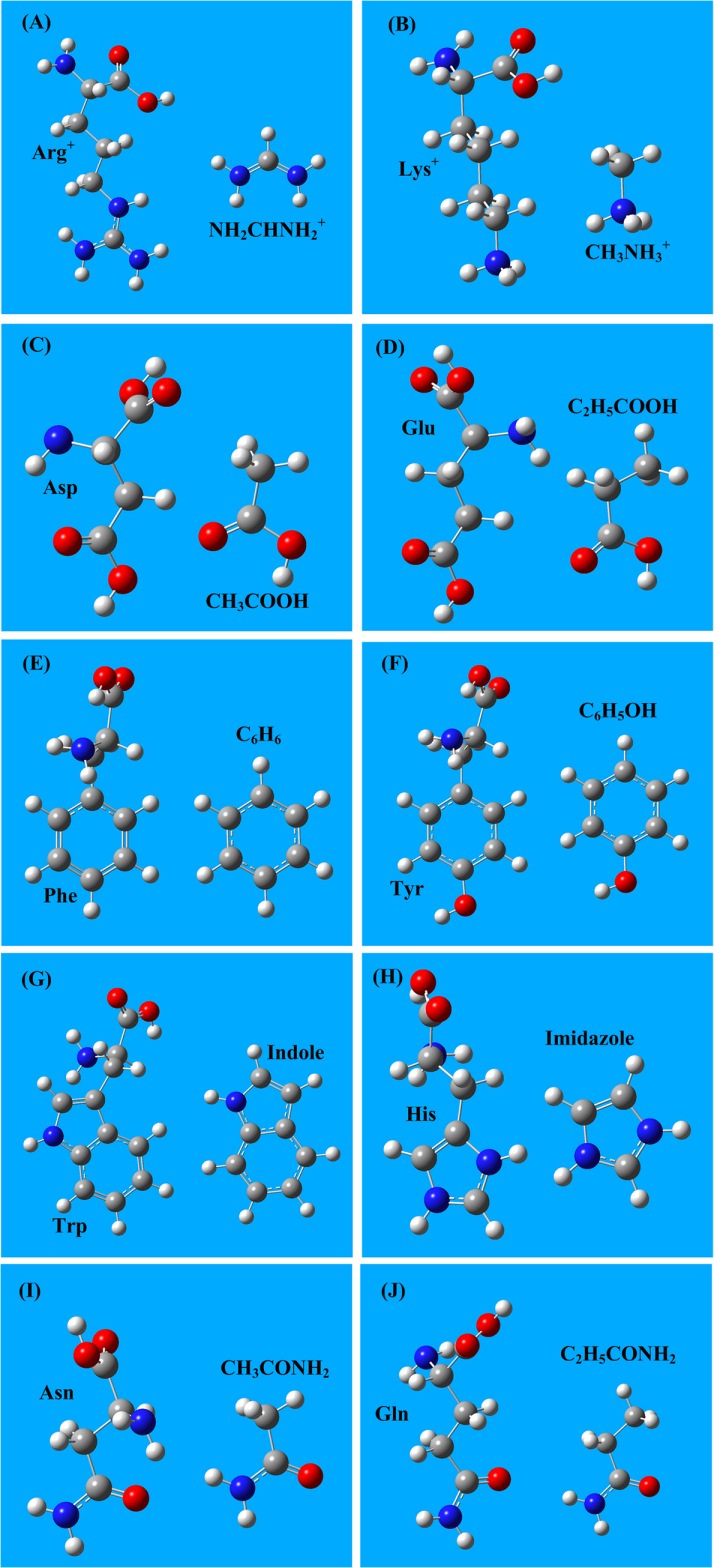
The side chain structures of the 8 amino acids involved in the salt-bridge and cation-π interactions. **A)** The protonated Arg^+^ is simplified as the NH_2_CHNH_2_^+^ cation. **B)** The protonated Lys^+^ is simplified as the CH_3_NH_3_^+^ cation. **C)** The side chain of acidic amino acid Asp is represented by CH_3_COOH. **D)** The side chain of acidic amino acid Glu is represented by C_2_H_5_COOH. **E)** The side chain of the aromatic amino acid Phe is C_6_H_6_. **F)** The side chain of the aromatic amino acid Tyr is C_6_H_5_OH. **G)** The side chain of the aromatic amino acid Trp is the indole ring. **H)** The side chain of the aromatic amino acid His is the imidazole group. **I)** The side chain of the amino acid Asn is CH_3_CONH_2_. **J)** The side chain of the amino acid Gln is C_2_H_5_CONH_2_.

In the energy calculations of aa side chain interactions, the amino acids are simplified to only their side chains. All monomer structures of amino acids and their side chains are shown in **Fig 1.**

In this study the aa side chain interaction energies are defined as the energy difference Δ*E*(a-b) between the energy *E*(a-b) of the aa pair-complex a-b and the energy summation *E*(a)+*E*(b) of the two amino acid monomers a and b,

ΔE(a−b)=E(a−b)−[E(a)+E(b)] (1)

Positive values of Δ*E*(a-b) represent repulsive interactions, while negative values describe attractive interactions.

Calculations performed using the state-of-the art quantum chemical method CCSD(T) (coupled-cluster with single, double and partial triple excitations) [31–36] are extremely expensive and CPU-time consuming. Alternatively, the post Hartree-Fock method MP2 (a second order perturbation theory method) [37–39] can provide higher accuracy than H-F and DFT methods [40–50] and uses much less CPU-time than CCSD(T) methods [31–36]. In this study, all aa side chain monomer structures are optimized using the MP2 method [37–39] with a 6–311+G(d,p) basis set [51]. The geometries and energies of the interacting aa side chain pairs are calculated and optimized at the MP2/6–311+G(d,p) level. Then more accurate interaction energies of aa side chain pairs are calculated using the state-of-the art CCSD(T)/6–311+G(d,p) method [31–36] at the optimized structures. The aa side chain interaction energies in aqueous solutions are calculated using the polarizable continuum model (PCM) [52–55] method. All calculations are performed using the Gaussian 09 software package [56] at TH-1 A super computer center (www.nscc-tj.gov.cn).

## Results

The calculation results of three types of strong aa interactions (salt bridge, cation-π, and amide bridge) are reported and summarized in the tables and figures. The factors that affect the interactions are described and analyzed.

### Amino acid salt bridge interactions

An aa salt bridge interaction is the interaction between the base of an alkaline amino acid and the root of an acidic amino acid [57–59]. In the 20 natural amino acids there are three alkaline amino acids (Arg, Lys and His) and two acidic amino acids (Glu and Asp). The acidic dissociation constants of the above 5 amino acids [58] are listed in **Table 1**.

**Table 1:** The pK_a_ of the three alkaline amino acids (Arg, Lys and His) and the two acidic amino acids (Glu and Asp) [60].

Amino acid	Code	pK_a_
Arginine	Arg (R)	12.48
Lysine	Lys (K)	10.53
Histidine	His (H)	6.00
Glutamic acid	Glu (E)	4.25
Aspartic acid	Asp (D)	3.65

**Fig 2:**
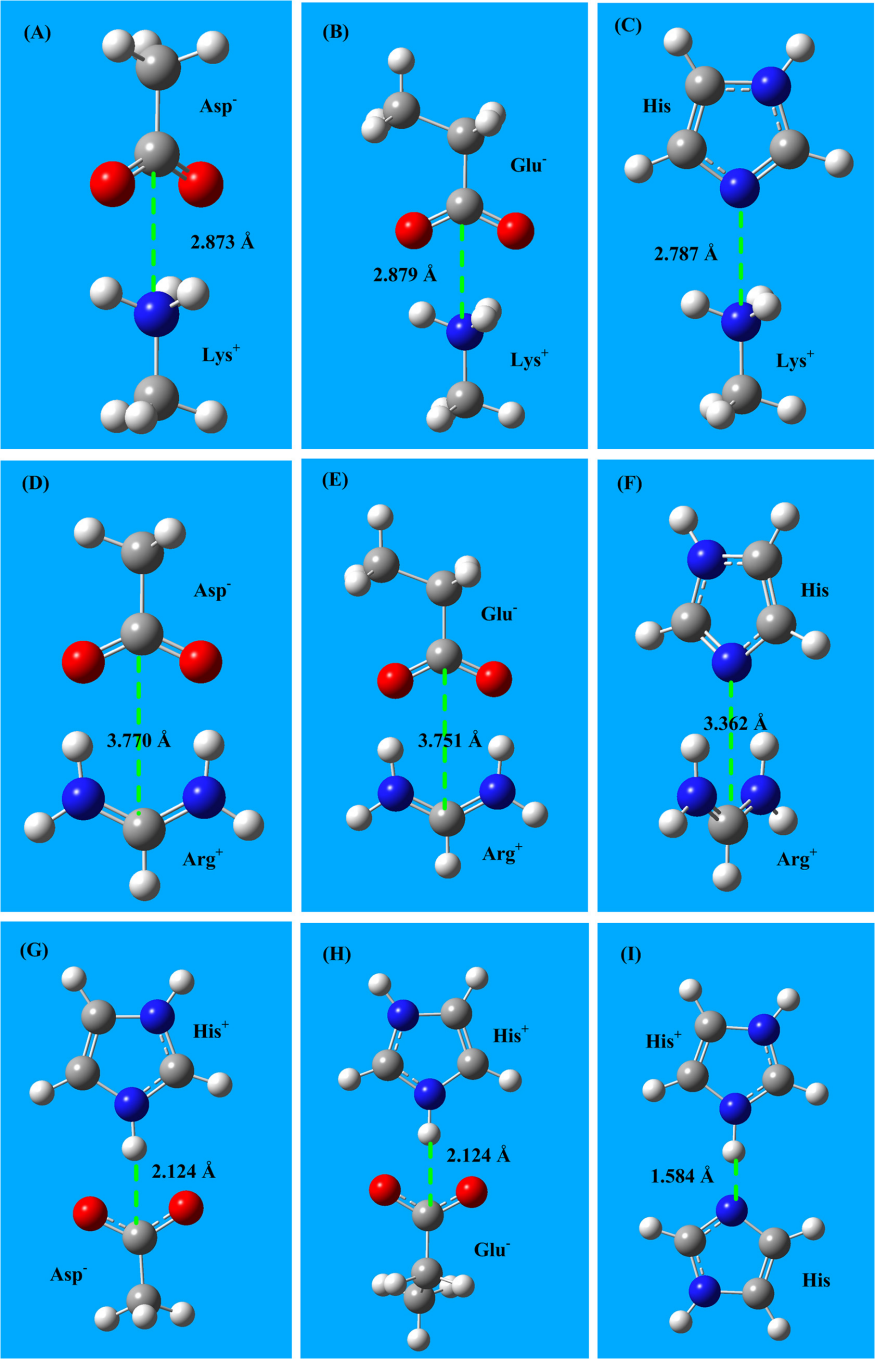
The salt-bridge interaction structures between three amino acid cations (Arg^+^ Lys^+^, and His^+^) and three acidic amino acids (Asp^-^, Glu^-^, and His). **A)** The salt-bridge structure of Lys^+^–Asp^-^. **B)** The salt-bridge structure of Lys^+^–Glu^-^. **C)** The salt-bridge structure of Lys^+^–His. **D)** The salt-bridge structure of Arg^+^–Asp^-^. **E)** The salt-bridge structure of Arg^+^–Glu^-^. **F)** The salt-bridge structure of Arg^+^–His. **G)** The salt-bridge structure of His^+^–Asp^-^. **H)** The salt-bridge structure of His^+^–Gln^-^. **I)** The salt-bridge structure of His^+^–His. The energies of aa salt bridge interactions are in the range -90 to -520 kJ/mol.

In the aa salt-bridge interaction calculations the two alkaline amino acids (Arg and Lys) are in the protonated form (cations Arg^+^ and Lys^+^). The two acidic amino acids (Asp and Glu) are deprotonated (anions Glu^-^ and Asp^-^). Histidine (His) is a very weak alkaline amino acid having a pK_a_ of 6.08, which means that in proteins, histidine could appear in both the neutral form (His) and in the protonated form (His^+^). In this study salt bridge interaction energies are calculated using the MP2/6–311+G(d,p) method followed by the CCSD(T)/6–311+G(d,p) method. The interaction distances are fully optimized using MP2 calculations, and these optimized geometries are used in the subsequent CCSD(T) calculations. The interaction structures of the six aa salt-bridge pairs are shown in **Fig 2**, and the interaction energies and bond lengths are listed in **Table 2**.

**Table 2:** Salt-bridge interaction energies between the two alkaline amino acids (Arg and Lys) and the three acidic amino acids (Glu, Asp and His).

	MP2	/6–311+	G(d,p)		CCSD(T)	/6–311+	G(d,p)	
Interaction	Gaseous	phase	Aqueous	phase	Gaseous	phase	Aqueous	phase
pairs	Δ*E*_s-b_	Bond	Δ*E*_s-b_	Bond	Δ*E*_s-b_	Bond	Δ*E*_s-b_	Bond
	kJ/mol	Å	kJ/mol	Å	kJ/mol	Å	kJ/mol	Å
Asp^-^–Lys^+^	-494.3	2.884	-33.17	2.999	-497.7	2.873	-37.64	2.992
Asp^-^–Arg^+^	-526.6	3.778	-70.58	3.921	-528.1	3.770	-72.68	2.922
Glu^-^–Lys^+^	-496.3	2.887	-38.37	2.997	-498.5	2.879	-40.35	2.950
Glu^-^–Arg^+^	-523.2	3.780	-71.45	3.919	-525.34	3.751	-73.25	3.902
His–Lys^+^	-89.10	2.793	-11.50	2.901	-93.13	2.787	-15.93	2.893
His–Arg^+^	-106.7	3.770	-28.38	3.827	-111.9	3.751	-37.07	3.845
His^+^–Asp^-^	-474.0	2.124	-59.25	3.564	-476.2	2.102	-62.52	3.586
His^+^–Glu^-^	-473.1	2.124	-59.13	3.562	-475.9	2.104	-62.15	3.581
His^+^–His	-149.2	1.584	-24.87	2.563	-153.2	1.552	-26.92	2.667

In the gaseous phase the salt-bridge interaction energies (-400 ∼ -500 kJ/mol) of Asp^-^ and Glu^-^ are in the range of chemical bonds. These energies are far beyond molecular interaction energies, which usually are less than 100 kJ/mol. However, the salt-bridge energies (-90 -110 kJ/mol) of His are smaller than those of the Asp^-^ and Glu^-^, because the histidine is in neutral form (His), not in anionic form. In aqueous solutions, the aa salt-bridge energies (-20 -70 kJ/mol) decrease almost 80%, however still stronger than other molecular interaction types (e.g., van der Waals interactions, electrostatic interactions, and hydrogen bonds).

The salt-bridge energies of Arg^+^ are larger than that of Lys^+^ because Arg^+^ has a higher pK_a_ value than Lys^+^ (12.00 and 10.50, respectively). On the other hand, Arg^+^ has two equivalent NH_2_ groups that may interact with the two oxygen atoms in the carboxyl groups (COO^-^) of Asp^-^ and Glu^-^, forming very strong salt-bridge bonds, as shown in **Fig 2D** and **Fig 2E**. In the Arg^+^–His salt-bridge structure (**Fig 2F**), the π-plane of imidazole and the π-plane of NH_2_CHNH_2_^+^ are oriented perpendicularly.

The salt-bridge energies of Asp^-^ are slightly larger than that of Glu^-^ because the pK_a_ value of Asp^-^ is lower than that of Glu^-^ (3.90 and 4.30, respectively). In acidic solutions the aa salt-bridge may be broken, because Asp and Glu are weak acids and may be protonated at lower pH (pH<4.0). Histidine (His) is a unique amino acid that has a pK_a_ of 6.08 [60] and can play the role of either proton donor or acceptor. Therefore, the salt-bridge interactions of histidine are easily affected by many factors. Please refer to reference [4] for details.

The aa salt-bridge energies calculated using CCSD(T) are very similar to those calculated using MP2. In general, the salt-bridge energies provided by CCSD(T) are 2 kJ/mol stronger than those obtained with the MP2 method.

### Amino acid cation-π interactions

**Fig 3:**
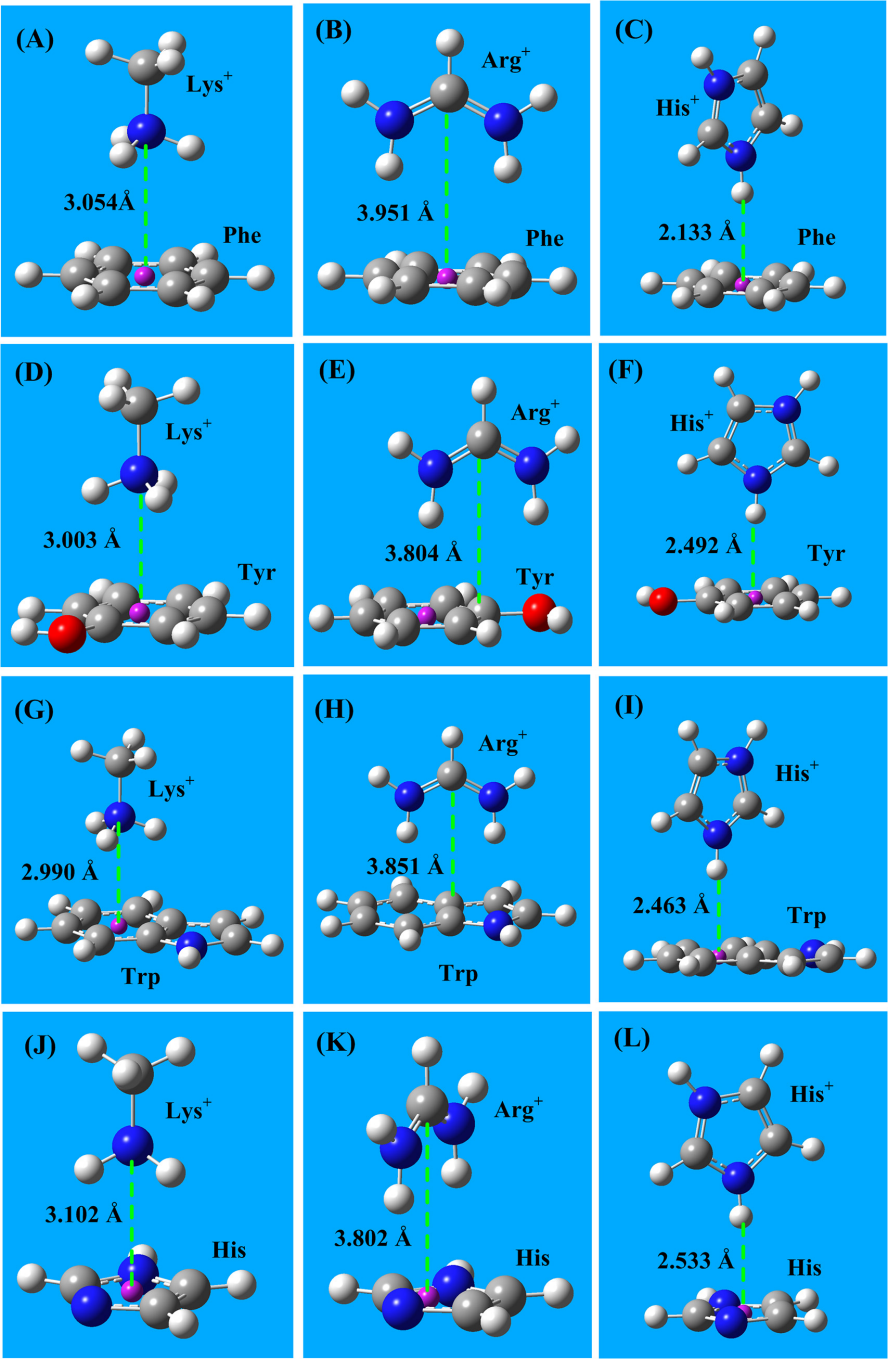
The cation-π interaction structures between three cations (Arg^+^, Lys^+^, and His^+^) and four aromatic amino acids (Phe, Tyr, Trp, and His). **A)** The cation-π interaction structure of Lys^+^–Phe. **B)** The cation-π interaction structure of Arg^+^–Phe. **C)** The cation-π interaction structure of His^+^–Phe. **D)** The cation-π interaction structure of Lys^+^–Tyr. **E)** The cation-π interaction structure of Arg^+^–Tyr. **F)** The cation-π interaction structure of His^+^–Tyr. **G)** The cation-π interaction structure of Lys^+^–Trp. **H)** The cation-π interaction structure of Arg^+^–Trp. **I)** The cation-π interaction structure of His^+^–Trp. **J)** The cation-π interaction structure of Lys^+^–His. **K)** The cation-π interaction structure of Arg^+^–His. **L)** The cation-π interaction structure of His^+^–His. The energies of aa cation-π interactions are in the range -50 to -85 kJ/mol.

In proteins, the aa cation-π interactions are the interactions between protonated amino acids (cations Arg^+^, Lys^+^ and His^+^) and aromatic amino acids (Phe, Tyr, Trp and His). From a physical perspective, cation-π interactions are the interactions between cations and the π-electron density of conjugated molecules (or groups), including electrostatic contributions and orbital coordinate contributions [61–66]. In cation-π interactions, the cation perpendicularly points to the conjugate π-plane, and the most stable interaction distances are 2.5 to 3.5 Å. In proteins, histidine may frequently change between the neutral (His) and protonated (His^+^) form. The geometries of the cation-π interactions between three cations (Arg^+^, Lys^+^, and His^+^) and four aromatic amino acids (Phe, Tyr, Trp, and His) are shown in **Fig 3**.

The cation-π interactions are point (cation) to π-plane interactions that allow more possible structural conformations, and possess broader interaction range than hydrogen bond interactions. In heteroaromatic π-groups, such as the side chains of Tyr, His, and Trp, the potential energy surface of cation-π interactions along the π-plane is very complex.

The interaction energies and the bond lengths of cation-π interactions, calculated using MP2/6–311+G(d,p) and CCSD(T)/6–311+G(d,p) methods, are listed in **Table 3**. The energies of aa cation-π interactions are in the range of -40 to -85 kJ/mol, which are much stronger than that of typical hydrogen bonds (∼ -20 kJ/mol). In aqueous solutions, the cation-π interactions are weakened by the high dielectric constant of water; however, the decrease in strength of cation-π interactions in solution is smaller than that of salt-bridge interactions in solution, because the cation-π interactions contain more orbital coordinate contributions, which are only mildly influenced by solvent effects. The cation-π interaction energies obtained using the CCSD(T) method are approximately 10 kJ/mol stronger than that obtained using the MP2 method.

**Table 3:** Cation-π interaction energies between the three cationic amino acids (Arg^+^, Lys^+^ and His^+^) and the four aromatic amino acids (Phe, Try, Trp, and His).

	MP2	/6–311+	G(d,p)		CCSD(T)	/6–311+	G(d,p)	
Interaction	Gaseous	phase	Aqueous	phase	Gaseous	phase	Aqueous	phase
pairs	Δ*E*_s-b_	Bond	Δ*E*_s-b_	Bond	Δ*E*_s-b_	Bond	Δ*E*_s-b_	Bond
	kJ/mol	Å	kJ/mol	Å	kJ/mol	Å	kJ/mol	Å
Lys^+^–Phe	-51.18	3.084	-9.107	3.102	-60.28	3.062	-12.61	3.184
Lys^+^–Tyr	-51.31	3.071	-9.154	3.092	-62.80	3.001	-13.78	3.005
Lys^+^–Trp	-71.53	2.990	-12.678	3.043	-86.41	2.903	-16.75	2.990
Lys^+^–His	-39.99	3.072	-7.142	3.101	-50.28	3.051	-11.78	3.083
Arg^+^–Phe	-50.85	3.988	-5.319	4.262	-60.51	3.951	-14.96	3.970
Arg^+^–Tyr	-52.29	3.836	-3.911	4.070	-67.87	3.804	-18.58	4.013
Arg^+^–Trp	-76.86	3.902	-5.102	4.116	-87.69	3.851	-24.55	3.952
Arg^+^–His	-45.81	3.851	-3.992	4.048	-54.48	3.802	-13.51	3.981
His^+^–Phe	-48.85	2.988	-6.324	3.324	-52.63	2.953	-13.84	2.206
His^+^–Tyr	-5034	2.864	-4.821	3.362	-57.78	2.838	-16.85	2.170
His^+^–Trp	-66.68	2.954	-6.212	3.318	-72.96	2.802	-22.56	2.112
His^+^–His	-44.81	2.981	-4.546	3.648	-50.84	2.862	-12.15	2.533

### Amide bridge interactions

**Fig 4:**
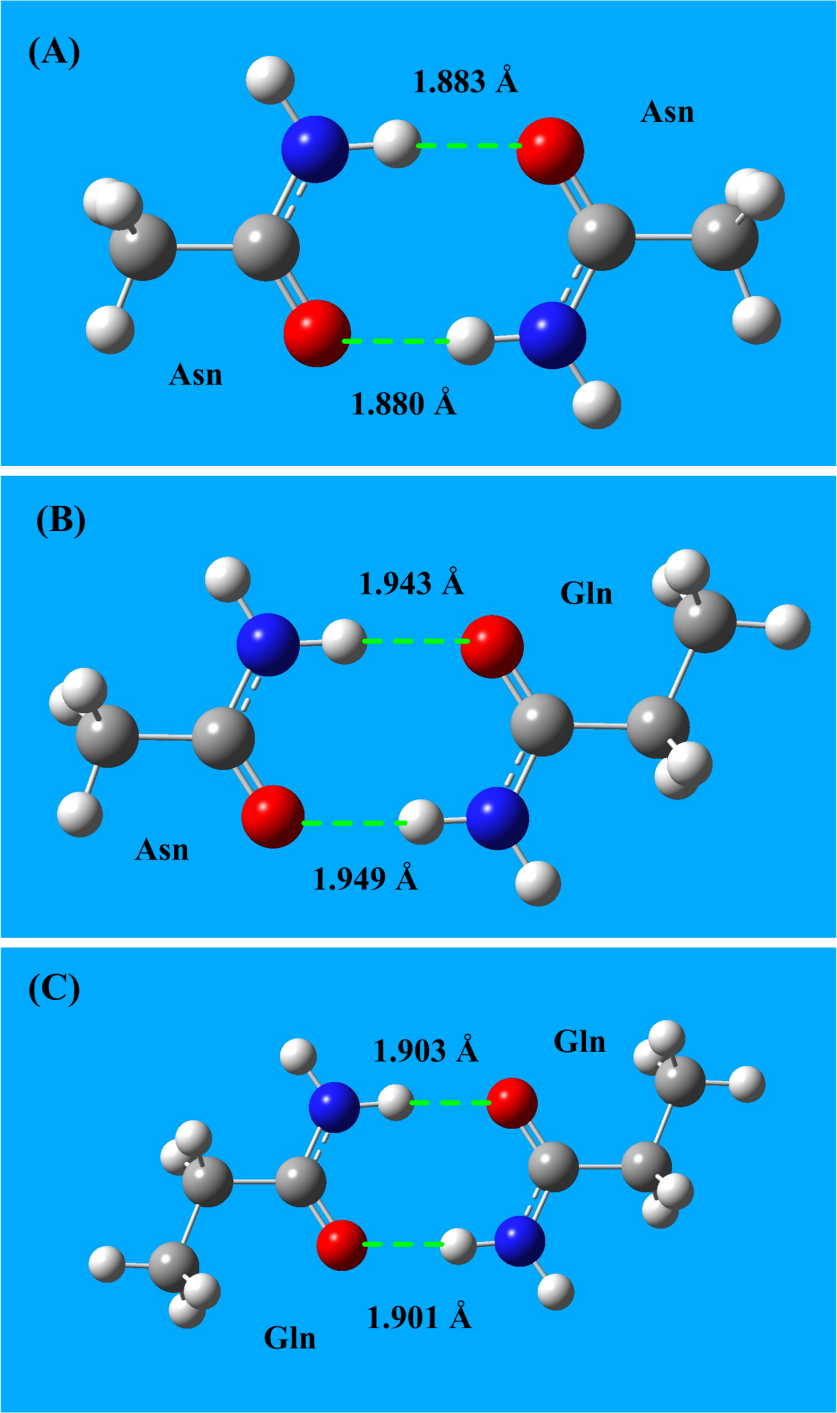
Amide bridge interactions between amino acids Asn and Gln. **A)** Interaction structure of amide bridge for the Asn-Asn interaction. **B)** Interaction structure of amide bridge for the Asn-Gln interaction. **C)** Interaction structure of amide bridge for the Gln-Gln interaction. The energies of aa amide bridge interactions are in the range -65 to -70 kJ/mol.

The two amide-containing amino acids, Asn and Gln, possess both a partially positively charged NH_2_ group and a partially negatively charged C = O group, as shown in **Fig 1I** and **Fig 1J**. Therefore, two amide-containing amino acids can form an amide bridge. The structures of three types of amide bridges (Asn-Asn, Asn-Gln, and Gln-Gln) are shown in **Fig 4**, and the interaction energies of amide bridges are listed in **Table 4**. The energies of aa amide bridge interactions are in the range of -65 to -70 kJ/mol, which is three times higher than typical hydrogen bond energies (∼-20 kJ/mol).

**Table 4:** Amide bridge interaction energies between the two amide amino acids (Asn and Gln).

	MP2	/6–311+	G(d,p)		CCSD(T)	/6–311+	G(d,p)	
Interaction	Gaseous	phase	Aqueous	phase	Gaseous	phase	Aqueous	phase
pairs	Δ*E*_s-b_	Bond	Δ*E*_s-b_	Bond	Δ*E*_s-b_	Bond	Δ*E*_s-b_	Bond
	kJ/mol	Å	kJ/mol	Å	kJ/mol	Å	kJ/mol	Å
Asn-Asn	-61.90	1.884	-9.407	2.102	-69.28	1.862	-13.61	1.984
Asn-Gln	-52.57	1.943	-9.254	2.292	-67.80	1.881	-11.78	2.005
Gln-Gln	-59.07	1.902	-12.766	2.183	-68.41	1.863	-12.75	1.990

## Applications

Salt-bridge, cation-π, and amide bridge interactions frequently occur in protein-protein and protein-drug interactions, and often play important roles in these interactions. A solid understanding of these three types of interactions is greatly helpful for the rational design of drugs that target host proteins. Three examples of applications are presented in this section.

### The binding sites of amantadine in M2 proton channel

**Fig 5:**
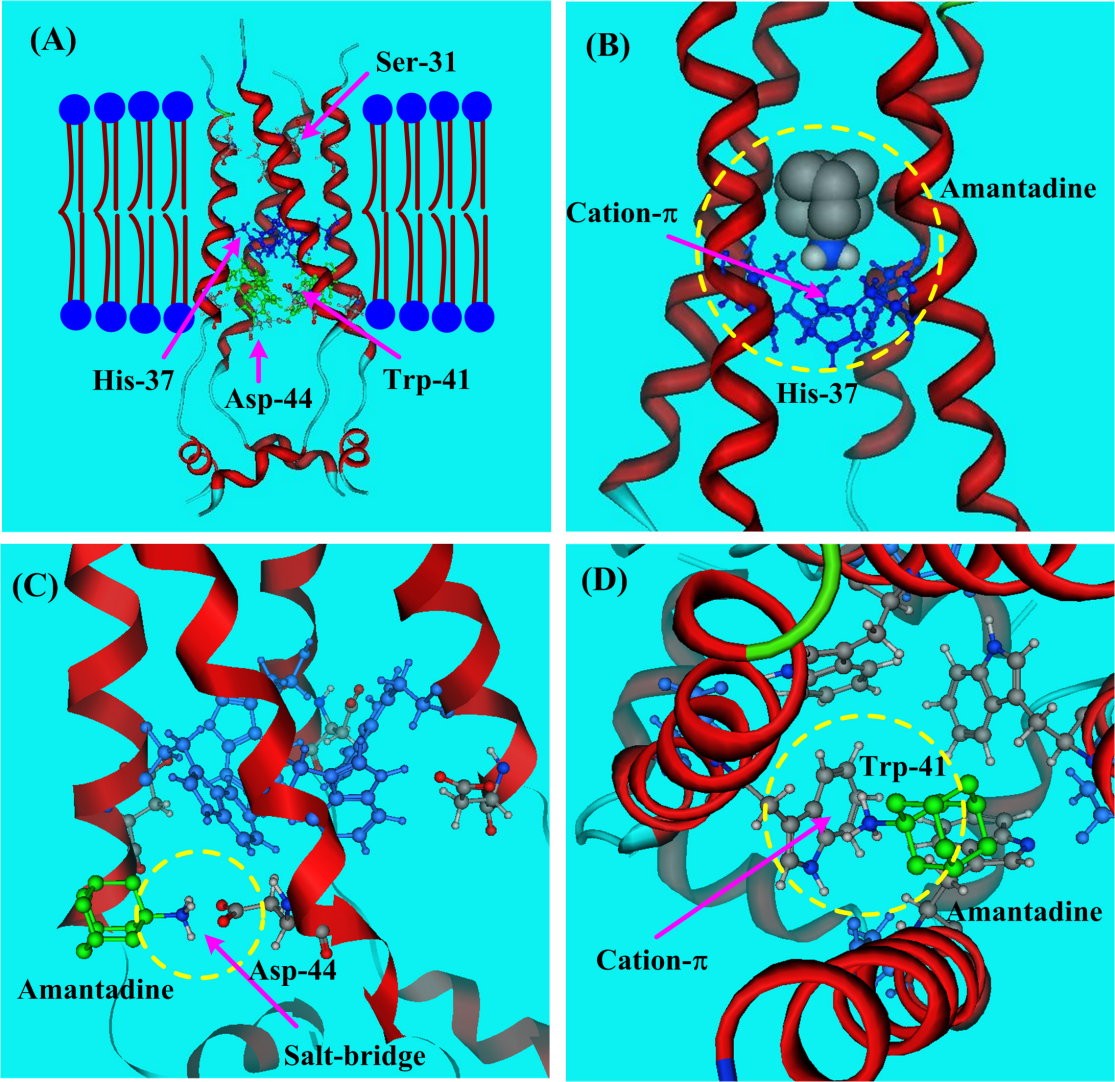
The structure of the M2 proton channel of influenza A virus (PDB code: 2RLF). **A)** Possible binding sites (Ser-31, His-37, Trp-41, and Asp-44) of amantadine. **B)** The cation-π interaction between amantadine and His-37 in M2 proton channel. **C)** The salt-bridge interaction between amantadine and Asp-44. **D)** The cation-π interaction between amantadine and Trp-41.

The binding sites of amantadine in the M2 proton channel [26,27] of the influenza A virus has drawn great attention and stimulated broad discussion among many authors [67–70]. The structure of the channel and four of its particularly important residues (Ser-31, His-37, Trp-41, and Asp-44) is shown in **Fig 5A**. The general conclusion of previous studies [67,68] is that the amantadine binding location could be inside the pore of the M2 channel or outside the M2 channel on a lipid-facing side, depending on the ligand concentration and the dynamic steps [67,68].

When the amantadine enters the channel pore, the best binding sites are the four His-37 residues, which form stable cation-π interactions with the amine group (NH_3_^+^) of amantadine. When the amantadine ligand binds to the outside of the M2 channel near the gate of the channel, a favored binding site is the Asp-44, where the NH_3_^+^ of amantadine and the anionic Asp^−^-44 form a very stable salt-bridge, as shown in **Fig 5C**. The salt-bridge interaction energy between Asp^-^ and amantadine could reach -70 to -500 kJ/mol, depending on the solvation environment.

The M2 protein is a proton channel. In acidic conditions, the salt bridge between amantadine and Asp-44 is at risk of being broken. When it does break, the amantadine may come into the channel and form a cation-π bond with Trp-41. According to MP2/6–311+G(d,p) calculations, the cation-π interaction energy between amantadine and the Trp-41 is -78.70 kJ/mol [69] in the gaseous phase. However, in an aqueous solution, this cation-π interaction energy may decrease to -13.27 kJ/mol. In **Fig 5,** the salt-bridge and cation-π interactions are indicated by yellow cycles.

Currently, in almost 95% of the cases where the influenza A virus is encountered, the virus has the S31N mutation that confers drug resistance. There is a silver lining to the nearly ubiquitous presence of this mutated Asn-31residue. It may provide a good binding site for inhibitor design; new inhibitors could bind at Asn-31 through amide bridge interactions.

### Inhibitor design targeting neuraminidase of influenza A virus

**Fig 6:**
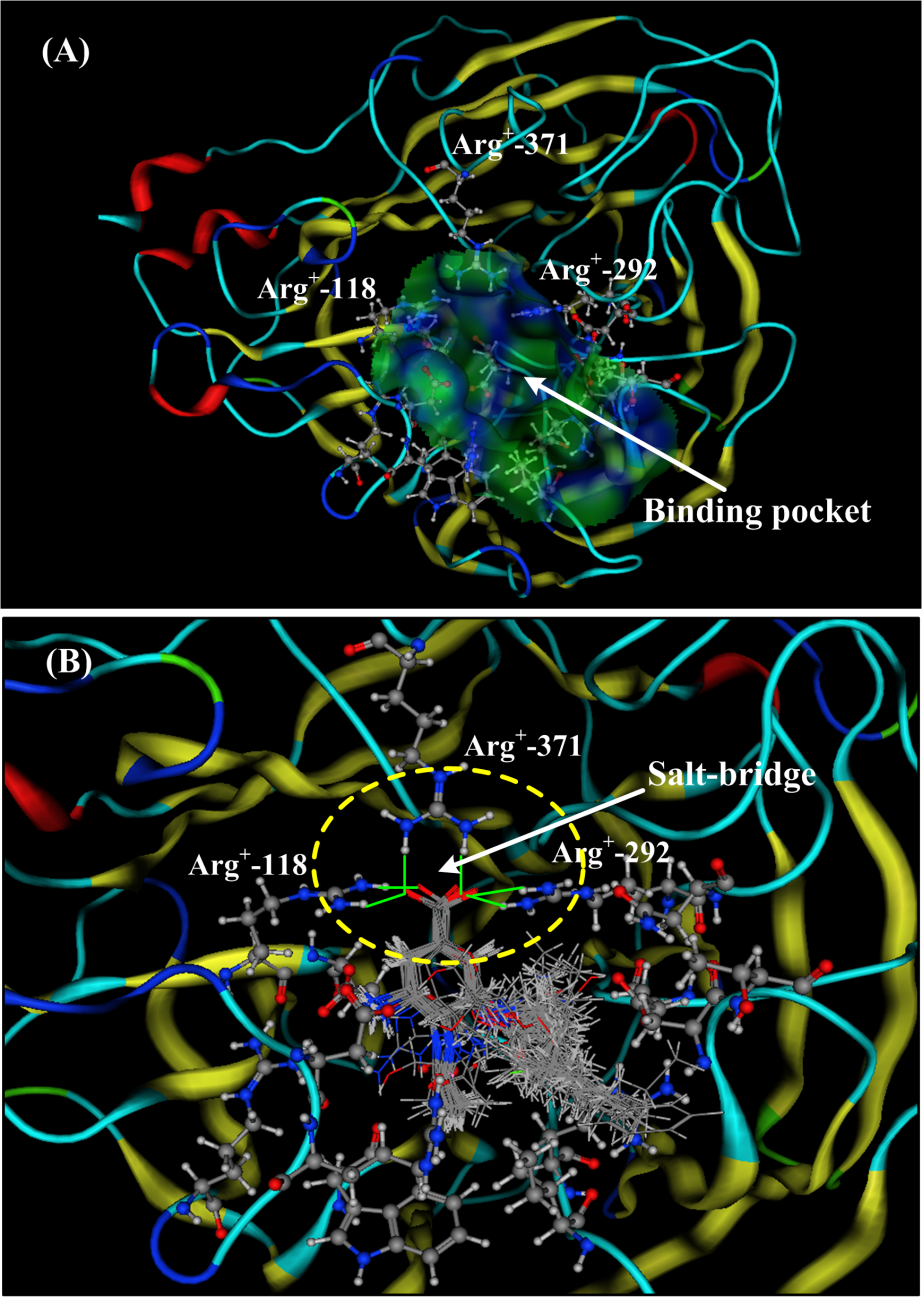
The neuraminidase (NA) structure of influenza A virus (PDB code: 1F8B). **A)** The binding pocket for ligands in the neuraminidase (NA) of influenza A virus. The hydrophobic pocket consists of 17 residues (Ala118, Leu119, Asn151, Ser152, Leu156, Pro178, Ser198, Met222, Asp224, Ser246, Val247, Trp276, Tyr292, Ile294, Gln371, Leu406, and Phe425). **B)** The docking structure and salt-bridge interactions of 49 drugs (or ligands) in the binding pocket of neuraminidase (1F8B). There is a formal salt-bridge between the carboxyl group (–COO^-^) of the ligands and the residue Arg^+^-371. The other two arginine residues (Arg^+^-118 and Arg^+^-292) are on either side of the carboxyl group (–COO^-^), forming two partial salt-bridge bonds with the oxygen atoms of the carboxyl group. The three salt-bridge bonds are indicated by yellow circles.

In the design of drugs that target host proteins, salt-bridge interactions may play a very important role and often account for a large portion of the binding free energies. Oseltamivir and zanamivir were designed based on the neuraminidase (NA) structure (1F8B) of the influenza A virus [70]. The structure of NA 1F8B is shown in **Fig 6A** and features a ligand located in a pocket consisting of 17 residues (Ala118, Leu119, Asn151, Ser152, Leu156, Pro178, Ser198, Met222, Asp224, Ser246, Val247, Trp276, Tyr292, Ile294, Gln371, Leu406, and Phe425). In ref [71], 49 drugs and ligands (including oseltamivir and zanamivir) are aligned and docked with the neuraminidase (1F8B), as shown in **Fig 6B**.

According to the docking structure, all 49 of the ligands possess the same pharmacophore, the carboxyl group (COO^-^), which is surrounded by three arginine residues (Arg^+^-118, Arg^+^-292, and Arg^+^-371). Between the pharmacophore (COO^-^) and the Arg^+^-371 residue, there is a very strong salt-bridge bond, and the distances between the two oxygen atoms of the carboxyl group (COO^-^) and the two—NH_2_ groups of Arg^+^-371 are 1.72 Å and 1.63 Å, respectively. The interaction energy of the salt-bridge could be as high as-526 kJ/mol in the gaseous phase. The other two arginine residues (Arg^+^-118 and Arg^+^-292) are on either side of the carboxyl group (COO^-^), forming two partial salt-bridge bonds with the two oxygen atoms of the carboxyl group. The three salt-bridge bonds are indicated by yellow circles in **Fig 6B**.

### Binding site of amantadine in the HCV p7 ion channel

**Fig 7:**
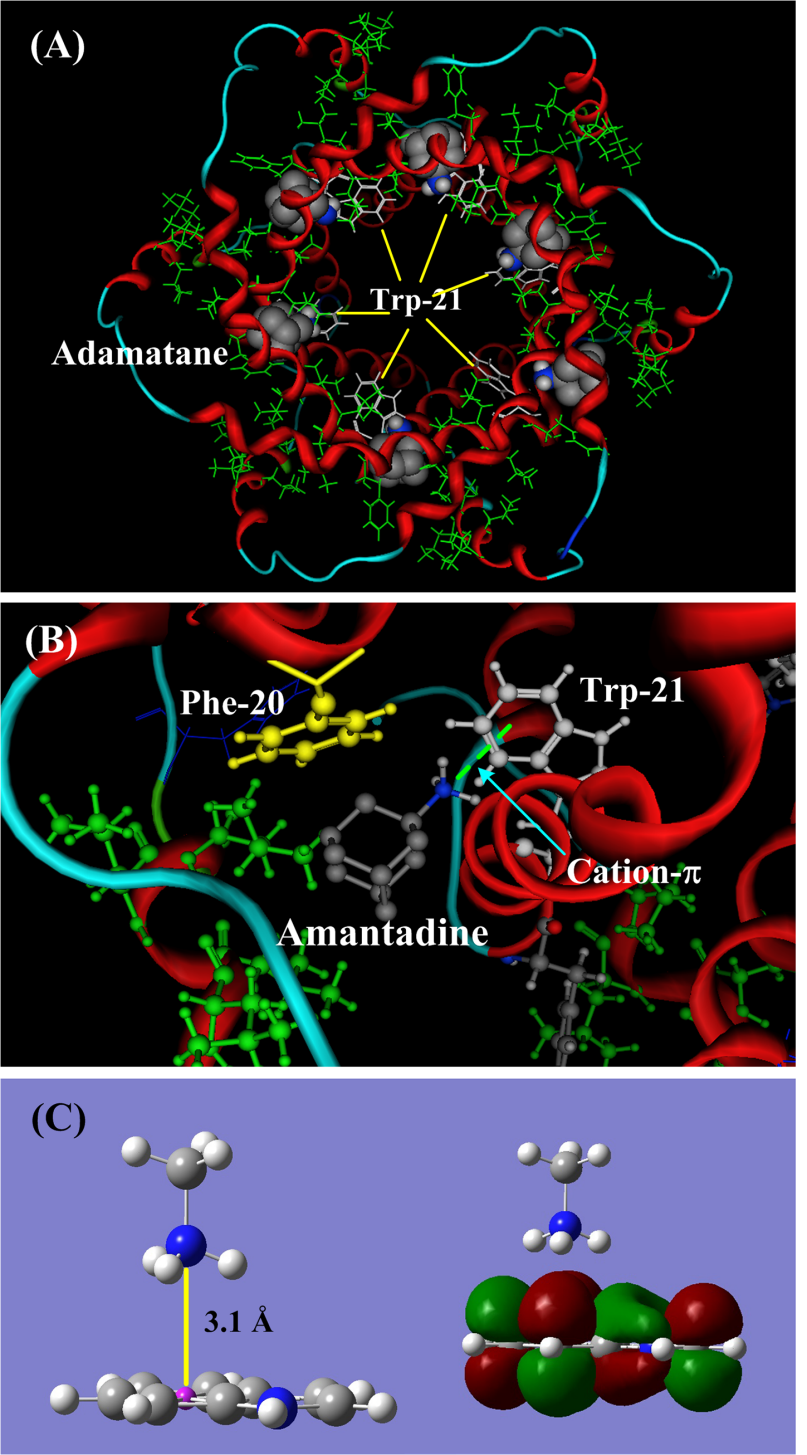
The structure and binding sites of amantadine in p7 ion channel (PDB code: 2M6X) of the hepatitis C virus (HCV). **A)** In the p7 channel, the ligand amantadine is in the six hydrophobic pockets consisting of Leu 52, Val 53, Leu55, and Leu 56 from H3 and Phe 20, Val 25, and Val 26 from H2. The hydrophobic residues are shown in green and Trp-21 is shown in white lines. **B)** The cation-π interaction between the NH_3_^+^ group of amantadine and the aromatic indole ring of Trp-21. When Leu20 is replaced by Phe20 (yellow color), the ammonium group (NH_3_^+^) of amantadine may shift its orientation and form a cation-π bond with Phe20. This mayreasonably explain the Leu20Phe mutation that confers drug resistance in some p7 channel subtypes. **C)** The molecular orbital (MO) of the cation-π interaction between the NH_3_^+^ group and the aromatic indole ring. The cation perpendicularly points to the π-plane.

The NMR solution structure of the p7 ion channel (PDB code: 2M6X) of the hepatitis C virus (HCV) was first solved by Chou and his colleagues [30]. In the p7 channel, there are six similar hydrophobic pockets between the peripheral and the pore-forming helices, consisting of Leu-52, Val-53, Leu-55, and Leu-56 from H3 and Phe-20, Val-25, and Val-26 from H2 [30]. The ligand amantadine is located in the hydrophobic pockets, as shown in **Fig 7A**. In the binding location described by Chou and colleagues, the pharmacophore group (NH_3_^+^) of the amantadine points to the aromatic indole ring of Trp-21, forming a stable cation-π bond (indicated by light green dished line), as shown in **Fig 7B**. The binding energy of the cation-π interaction could be -86.41 kJ/mol in the hydrophobic pocket, where the environment is similar to the gaseous phase.

In the structure of the p7 channel protein 2M6X, the Phe-20 (yellow ball-stick drawing in **Fig 7B**) is a mutated residue that replaces the original Leu-20, which is a drug-resistant mutation, as identified in clinical trials [72–74]. When the Leu-20 is replaced by Phe-20, the ammonium group (NH_3_^+^) of amantadine may reorient itself, allowing it to make a cation-π bond with Phe-20. The cation-π energy of the interaction of amantadine with Phe (-60.28 kJ/mol) is smaller than that of the interaction of amantadine with Trp (-86.41 kJ/mol). However, in some subtypes of the p7 channel the position of Phe-20 may be more favorable than that of Trp-21. This may give a reasonable explanation for the drug-resistant Leu20Phe mutation in some p7 channel subtypes.

## Discussion

Salt-bridge, cation-π, and amide bridge interactions could very frequently occur in proteins because 10 (Arg, Lys, Asp, Glu, Phe, Tyr, Trp, His, Asn and Gln) of the 20 natural amino acids can participate in at least one of these three types of interactions. The very high interaction energies of salt-bridge, cation-π, and amide bridge interactions make these interactions remarkably stronger than other molecular interaction types, such as hydrogen bonds, electrostatic interactions, and van der Waals interactions. These three types of interactions may not be properly described by molecular dynamics (MD) using the currently available force field parameters [63]. The interaction energies provided in this study are calculated from the optimized structures of amino acid side chains, which may be different from the actual interaction geometries.

In the natural world, the environments of proteins are very complex. The surfaces of proteins may be exposed to aqueous solution, while hydrophobic pockets inside the proteins may be in environments that, to a certain degree, more closely resemble the gaseous phase. However, the hydrophobic pockets are not completely equivalent to the gaseous phase because of the electrostatic fields formed by the polar groups of aa residues. In this study, the calculated energies in aqueous solution and in vacuo may reveal the limitations of the three types of interactions in different protein environments.

In the hydrophobic pockets, the salt-bridge interactions are very strong; however, they are weaker than the salt-bridge interactions in vacuo because of the electrostatic field produced by polar sidechains. Further, the salt-bridge bonds are weakened in aqueous solution and may be broken in acidic conditions. The cation-π and amide bridge interactions are less affected by solvation and pH conditions. Generally speaking, the aromatic amino acids (Phe, Tyr, Trp and His) are hydrophobic residues to a certain degree, and the amino acid cations (Arg^+^ Lys^+^ and His^+^) are hydrophilic residues. Therefore, cation-π interactions could occur at hydrophobic and hydrophilic interfaces. The salt-bridge, cation-π, and amide bridge interactions often play important roles in protein-protein, protein-peptide, and protein-ligand interactions.

## Conclusion

From this study some useful conclusions are summarized as follows. (1) Ten of the twenty natural amino acids are involved in the three types of strong interactions (salt-bridge, cation-π and amide bridge), which are much stronger than typical hydrogen bonds and often play important roles in protein-protein, protein-peptide, protein-ligand, and protein-DNA interactions. (2) The salt-bridge interactions between acidic (Glu^-^ and Asp^-^) and basic (Arg^+^, Lys^+^ and His^+^) amino acids are the strongest residue-residue interactions. However, salt-bridges may be weakened by solvation effects and may be broken by acidic conditions. (3) The cation-π interactions between protonated amino acids (Arg^+^, Lys^+^, and His^+^) and aromatic amino acids (Phe, Tyr, Trp and His) are 2.5 to 5-fold stronger than typical hydrogen bonds and are less affected by solvent and pH than are salt bridge interactions. The cation-π interactions could occur at the hydrophobic-hydrophilic interface. (4) Amide bridge interactions are special amino acid interactions that only occur between two amide amino acids (Asn and Gln), and these interactions are three times stronger than typical hydrogen bonds and less affected by pH.
